# Loneliness and its concomitants among older adults during the COVID-19 pandemic

**DOI:** 10.1017/S1041610220003476

**Published:** 2020-09-14

**Authors:** Maya Frenkel-Yosef, Ruth Maytles, Amit Shrira

**Affiliations:** 1 The Nini Czopp Association, Netanya 4250212, Israel; 2 Interdisciplinary Department of Social Sciences, Bar-Ilan University, Ramat-Gan 5290002, Israel Email: amit.shrira@biu.ac.il

Due to the COVID-19 crisis, older adults may face with what some have called a “loneliness epidemic” (Palgi *et al*., 2020). Complications from loneliness can include morbidity and death (Jeste *et al*., 2020). Hence, this study aimed to identify older adults who feel lonely during the pandemic.

Although COVID-19-related loneliness was actually lower among older compared to young adults (Losada-Baltar *et al*., 2020), pre-pandemic data suggest that loneliness is highest among the old-old (Beam and Kim, 2020). Beyond age and physical health (Jeste *et al*., 2020), negative views on aging (VoA) were also related to higher COVID-19-related loneliness (Losada-Baltar *et al*., 2020). Nevertheless, positive VoA – potentially reinforcing psychosocial resources (Schwartz *et al*., 2020) – are underexplored. Moreover, while psychological distress is a known correlate of loneliness (Palgi *et al*., 2020), less is known about common features during self-isolation, such as interaction via available means (phone, video, and face-to-face) or engagement in daily activities.

We hypothesized that loneliness would be highest among the oldest, those having medical conditions, more negative and less positive VoA, reduced interaction, and low activity engagement.

The sample included 295 older adults (mean age = 75.73, range 60–94, 68.5% women) located across Israel between April 23 and June 17, 2020, through contact lists provided by organizations related to older adults, and interviewed face-to-face, by phone, or requested to complete a web-based questionnaire when possible after providing informed consent to procedures approved by the ethics committee in Bar-Ilan University.

Background characteristics included age, gender, education, financial and marital status, number of children, and place of residence. COVID-19 exposure and medical conditions were reported as well.

Loneliness was assessed with the 3-item version of the UCLA Loneliness Scale (Hughes *et al*., 2004) (*α* = 0.91). VoA was assessed with the 12-item Attitudes to Aging Questionnaire (AAQ; Laidlaw *et al*., 2018) referring to three attitudes: psychological loss (*α* = 0.76), physical change (*α* = 0.64), and psychological growth (*α* = 0.79). Psychological distress was assessed via four items assessing anxiety and depressive symptoms (Kroenke *et al*., 2003, 2007) (*α* = 0.84). Interpersonal interactions were assessed by summing the number of contact persons (i.e. children, grandchildren, other family relatives, friends, and others) the participant had interacted with in recent weeks via phone, video, or face-to-face encounters. Activity engagement was assessed by the extent to which participants engaged in four activities (i.e. physical activity, leisure activities, daily planning, and executing plans) in previous weeks and whether it helped them cope with the pandemic (*α* = 0.76).

See Table 1 in the supplementary file for additional details about the sample, measures, and correlations between variables.

**Table 1.** Standard multiple regression predicting loneliness

	step 1		step 2		step 3		step 4		step 5		step 6	
	*B* (*SE*)	β	*B* (*SE*)	β	*B* (*SE*)	β	*B* (*SE*)	β	*B* (*SE*)	β	*B* (*SE*)	β
Age	0.02 (0.01)	0.14*	0.01 (0.01)	0.10	0.01 (0.01)	0.09	0.01 (0.01)	0.10	0.01 (0.01)	0.04	0.01 (0.01)	0.04
Education level	−0.05 (0.06)	−0.05	−0.05 (0.06)	−0.05	0.005 (0.06)	0.005	0.06 (0.06)	0.07	0.04 (0.06)	0.04	0.04 (0.06)	0.05
Self-rated financial status	−0.21 (0.09)	−0.15*	−0.19 (0.09)	−0.14*	−0.12 (0.08)	−0.08	−0.03 (0.08)	−0.02	−0.01 (0.08)	−0.01	0.001 (0.08)	0.001
Medical conditions	–	–	0.14 (0.06)	0.14*	0.09 (0.06)	0.09	0.09 (0.06)	0.09	0.10 (0.06)	0.09	0.10 (0.06)	0.09
AAQ – psychological loss	–	–	–	–	0.42 (0.09)	0.30***	0.26 (0.09)	0.19**	0.26 (0.09)	0.18**	0.26 (0.09)	0.19**
AAQ – physical change	–	–	–	–	−0.07 (0.10)	−0.05	−0.05 (0.09)	−0.04	−0.05 (0.09)	−0.03	−0.004 (0.09)	−0.003
AAQ – psychological growth	–	–	–	–	−0.01 (0.07)	−0.006	−0.001 (0.07)	−0.001	−0.01 (0.07)	−0.01	−0.005 (0.07)	−0.004
Psychological distress	–	–	–	–	–	–	0.16 (0.03)	0.34***	0.15 (0.03)	0.32***	0.13 (0.03)	0.29***
Phone interactions	–	–	–	–	–	–	–	–	0.04 (0.04)	0.06	0.04 (0.04)	0.05
Face-to-face interactions	–	–	–	–	–	–	–	–	−0.10 (0.04)	−0.13*	−0.10 (0.04)	−0.13*
Activity engagement	–	–	–	–	–	–	–	–	–	–	−0.27 (0.11)	−0.14*
*R* ^2^	0.06**		0.08***		0.17***		0.26***		0.28***		0.30***	

*Note*. *N* = 261. AAQ = Attitudes to Aging Questionnaire.

*p < 0.05, **p < 0.01, ***p < 0.001.

Loneliness was regressed on variables that were significantly correlated with it. Table 1 shows that older age and lower financial status, more medical conditions, and negative VoA (i.e. psychological loss, but not positive VoA, i.e. physical change and psychological growth) were related to higher loneliness in Steps 1 through 3, respectively. Psychological distress, less face-to-face interactions, and less activity engagement were associated with higher loneliness in Steps 4 through 6, respectively. Step 6 showed that the strongest concomitants of loneliness were negative VoA, psychological distress, fewer face-to-face interactions, and less activity engagement.

Although the oldest individuals reported higher levels of loneliness, the final model showed that loneliness was mainly associated with negative VoA, higher psychological distress, limited face-to-face interactions, and activity engagement.

Interestingly, only negative VoA were tied to increased loneliness in the final model. Adding to prior works (Losada-Baltar *et al*., 2020), it appears that negative VoA have a particularly harmful effect, whereas positive VoA may be less significant, with regard to loneliness. Moreover, few face-to-face interactions and an absence of regular activities were related to high loneliness; findings that join those linking absence of activities with distress (Fullana *et al*., 2020).

Our findings should be assessed in light of the study limitations: a convenience sample examined in a cross-sectional design without pre-pandemic measurement. With that said, we included a wide range of variables and interviewed individuals with limited access to or literacy in digital resources.

The findings suggest that face-to-face interactions are important, and therefore should be considered while maintaining necessary precautions. Loneliness might be further ameliorated by mitigating negative VoA and helping older adults plan and engage in activities.

## Supplementary material

For supplementary material accompanying this paper visit https://doi.org/10.1017/S1041610220003476. click here to view supplementary material
