# Supplementary material for: Loneliness and its concomitants among older adults during the COVID-19 pandemic
Source: Int Psychogeriatr. 2020 Sep 14:1–3. doi: 10.1017/S1041610220003476 (PMC7556899; doi:10.1017/S1041610220003476)
Supplement: Supplementary file 1 [file S1041610220003476sup001.docx]

**Loneliness and Its Concomitants among Older Adults during the COVID-19 Pandemic**

**Supplementary file**

Table 1 shows that loneliness was higher among those with more medical conditions, more negative views on aging, less positive views on aging, higher psychological distress, more phone interactions, less face-to-face interactions, and less activity engagement. Loneliness was also higher among older respondents, those with a lower education level and less favorable financial status.

| Table 1. *Means, SD, and Correlations for the Study Variables* | | | | | | | | | | | | | |
| --- | --- | --- | --- | --- | --- | --- | --- | --- | --- | --- | --- | --- | --- |
|  | *M/%* | *SD* | 1 | 2 | 3 | 4 | 5 | 6 | 7 | 8 | 9 | 10 | 11 |
| 1. Loneliness^1^ | 1.17 | 1.12 | - |  |  |  |  |  |  |  |  |  |  |
| 2. COVID-19 exposure^2^ | 1.11 | 1.22 | .10 | - |  |  |  |  |  |  |  |  |  |
| 3. Medical conditions^3^ | 1.17 | 1.07 | .19** | .11 | - |  |  |  |  |  |  |  |  |
| 4. AAQ-Psychological loss^4^ | 0.90 | 0.79 | .36*** | .08 | .17** | - |  |  |  |  |  |  |  |
| 5. AAQ-Physical change^5^ | 2.64 | 0.78 | -.25*** | -.04 | -.31*** | -.43*** | - |  |  |  |  |  |  |
| 6. AAQ-Psychological growth^6^ | 2.51 | 0.98 | -.14* | .07 | -.08 | -.34*** | .37*** | - |  |  |  |  |  |
| 7. Psychological distress^7^ | 1.94 | 2.46 | .44*** | .03 | .10 | .41*** | -.28*** | -.15* | - |  |  |  |  |
| 8. Phone interactions^8^ | 2.50 | 1.59 | .12* | .09 | .01 | .01 | -.01 | .04 | .05 | - |  |  |  |
| 9. Video interactions^8^ | 1.34 | 1.32 | .02 | .08 | -.05 | -.12* | .12* | .14* | -.06 | .21*** | - |  |  |
| 10. Face-to-face interactions^8^ | 1.74 | 1.46 | -.25*** | -.01 | -.13* | -.14* | .15* | .09 | -.16** | -.20*** | -.09 | - |  |
| 11. Activity engagement^9^ | 1.03 | 0.59 | -.29*** | -.05 | -.15* | -.19** | .32*** | .14* | -.31*** | -.09 | .12* | .12* | - |
| 12. Age | 75.73 | 8.06 | .18** | .07 | .33*** | .14* | -.18** | -.17** | .10 | .14* | -.03 | -.33*** | -.12* |
| 13. Gender (women) | 68.5 | - | .05 | -.08 | -.20** | .02 | .07 | .003 | .06 | .04 | -.05 | .03 | .05 |
| 14. Education level^10^ | 5.20 | 1.22 | -.15* | -.08 | -.11 | -.20** | .24*** | .08 | -.29*** | .04 | .08 | .16** | .15* |
| 15. Self-rated financial status^11^ | 3.88 | 0.79 | -.17** | -.02 | .17** | -.19** | .30*** | .05 | -.29*** | -.08 | .05 | .20*** | .21*** |
| 16. Marital status (married) | 66.1 | - | -.06 | -.01 | -.14* | -.08 | .03 | .04 | -.06 | -.02 | -.01 | .13* | .07 |
| 17. No. of children | 2.98 | 1.33 | -.07 | .08 | -.01 | -.10 | .10 | .13* | -.04 | .07 | .09 | .11 | .13* |
| 18. Place of residence^12^ (retirement home) | 8.2 | - | .05 | .17** | .15** | -.01 | -.03 | -.06 | .01 | .09 | -.15** | -.20*** | -.12* |

*Note*. *N* ranged 274-295. ^1^The average of three items referring to recent weeks rated on a 5-point scale (0=*not at all* to 4=*almost always*). ^2^The sum of exposure to six COVID-19 related events (e.g., being in isolation, having family members in isolation). ^3^The sum of chronic medical conditions suspected to increase the risk of death due to COVID-19 complications: cardiovascular disease, diabetes, chronic respiratory disease, hypertension, and cancer. ^4^The average of four items rated on a 5-point scale (0=*completely disagree* to 4=*completely* agree) referring to primarily seeing old age as a negative experience involving psychological and social losses (e.g., “Old age is a depressing time of life”). ^5^The average of four items rated on a 5-point scale (0=*completely disagree* to 4=*completely* agree) referring to being focused on health, exercise and the experience of aging (e.g., “My health is better than I expected for my age”). ^6^The average of four items rated on a 5-point scale (0=*completely disagree* to 4=*completely* agree) referring to positive gains that may have been a surprise about aging (e.g., “There are many pleasant things about growing older”). ^7^The sum of four items rated on a 4-point scale (0=*not at all* to 3=*almost every day*) referring to anxiety and depressive symptoms in the last two weeks. ^8^The sum of the number of contact persons (i.e., children, grandchildren, other family relatives, friends, and others) the participant had interacted with in recent weeks via phone, video or face-to-face encounters (possible range 0-5 for each interaction medium). ^9^The average of four items rated on a 3-point scale (0=*not engaging or engaging but engagement does not help*; 1=*engaging helps to some extent*; 2=*engaging greatly helps*) referring to activities in the prior weeks: physical activity, leisure activities (e.g., reading, listening to music), daily planning, and executing their plans for the day. ^10^Rated from 1, “*without formal education*” to 6, “*formal university degree*. ^11^Rated from 1, “*not good at all*” to 5, “*very good".* ^12^Rated as 0=community dwelling; 1=retirement home. AAQ= Attitudes to Aging Questionnaire. **p*<.05, ***p*<.01, ****p*<.001.
